# Tell me how much you move, I’ll tell you how much you’ll live. Editorial of the JONAFES I International Symposium of Physical Activity, Sport & Health

**DOI:** 10.2478/hukin-2014-0083

**Published:** 2014-11-12

**Authors:** André Luíz Gomes Carneiro

**Affiliations:** Chairman of the Scientific Committee

Every year, the JONAFES, National Journey of Physical Activity, Education and Health, promotes scientific initiatives in the State of Minas Gerais. Therefore, the Government of Minas Gerais, through the Secretary of State for Sport and Youth (SEEJ), supported this event in 2013, with the completion of the I International Symposium of Physical Activity, Sport & Health, highlighting the mission of sport and physical activity in the search for qualification by the professionals of physical activity and health.

Physical activity plays a foremost role with respect to health, starting to show its positive effects from childhood. In fact, the risk factors in adults may have their origin in childhood or adolescence and may be attenuated and / or prevented through regular physical activity at these ages. Taking into consideration that the relationship between physical activity and health is reflected in its protective effect by reducing the risk of developing degenerative and controllable illnesses, it might be expected that the same physical activity could decrease the mortality rate and increase longevity. Those benefits concern all age groups, however, physical activity does not have the same effect on all individuals nor do all individuals respond equally to the same stimuli. Thus, many diverse recommendations have been made within published guidelines regarding the quality and quantity of exercise for each of the populations and associated health problems (e.g. Garber et al., 2011). Consequently, by these factors, physical activity may be regarded as a prophylactic protection element of physical, psychological and social health.

Curiously, I would like to highlight one of Dr. Denise Taylor works with a very suggestive title: Physical activity is medicine for older adults (Taylor, 2014), which refers to the negative impact that inactivity produces on health, and because adults are less active than young people, the effort should be directed at increasing the levels of physical activity in this population. In other words, health searches for years of life at the same time that physical activity adds years to life.

The I International Symposium of Physical Activity, Sport & Health was attended by over 1300 professionals and students and more than 100 manuscripts were sent for evaluation. This special issue of the Journal of Human Kinetics aggregates the best papers evaluated by the scientific committee of the symposium.

In this special issue we highlight works of various areas of physical activity and health. For instance with postmenopausal women readers may consult the works on cardiorespiratory fitness and body composition (Moreira et al.); or the effects of resistance training and aquatic activities on cardiorespiratory and functional capacity (Novaes et al.); furthermore, the influence of resistance training on blood pressure in woman with metabolic syndrome (Cardoso et al.); and finally, the effect of an exercise program on bone mineral density in women with type 2 diabetes (Bello et al.). Still with type 2 diabetes, Sousa et al. studied the effects of resistance training on Uric Acid levels.

Regarding blood pressure and hypertension readers may consult a study about the acute effect of two different types of exercise programs on blood pressure (Mendes et al.), as well as another one with a blood flow restriction method in resistance exercise on hypertensive subjects (Araújo et al.)

In the field of body image there is a study attempting to verify the effects of different exercise programs on female body composition (Mendonça et al.) and a second one relating dance practice with body dissatisfaction and self-esteem in girls aged 9–15 (Monteiro et al.).

Relating respiratory function with the practice of Yoga readers may consult the paper of Bezerra et al.. Considering survivors of ischemic stroke, Aidar et al. present a paper over the effects of strength training on depression levels, and Monteiro et al. tried to verify how soccer practice effects lower limb amputees in their daily lives.

Some other authors presented school based intervention programs, one of those related to metabolic syndrome and Nutritional status (Cruz et al.), another one related to biological maturation, body morphology and physical performance (Freitas et al.), then a manuscript about programmed and self-selected physical activities and their effects on the performance levels (Neto et al.), and how gender effects pre-pubertal children after physical exercise intervention (Marta et al.).

In addition to these de Sousa et al. developed an automated step ergometer and present the respective data; Silveira et al., studied ergogenic supplementation among military subjects; Dias et al. explored different perceived exertion scales in walking or running with self-selected and imposed intensity; and finally, Cavaco et al. verified the short-term effects of complex training among young soccer players

As the chairman of the scientific committee of the I International Symposium of Physical Activity, Sport & Health I would like to acknowledge the participation and support of the following organizations: FUNORTE, FASI, SOEBRAS, Faculties Promove, Faculties Promove de Janaúba, INCISOH, CEIVA, FACIC, Faculties Kennedy and UTAD-CIDESD.

Wishes for good reading

